# TMEM106B expression is reduced in Alzheimer’s disease brains

**DOI:** 10.1186/alzrt247

**Published:** 2014-03-31

**Authors:** Jun-ichi Satoh, Yoshihiro Kino, Natsuki Kawana, Yoji Yamamoto, Tsuyoshi Ishida, Yuko Saito, Kunimasa Arima

**Affiliations:** 1 Department of Bioinformatics and Molecular Neuropathology, Meiji Pharmaceutical University, 2-522-1 Noshio, Kiyose, Tokyo 204-8588, Japan; 2 Department of Pathology and Laboratory Medicine, Kohnodai Hospital, National Center for Global Health and Medicine, 1-7-1 Kohnodai, Ichikawa, Chiba 272-8516, Japan; 3 Department of Laboratory Medicine, National Center Hospital, National Center of Neurology and Psychiatry, 4-1-1 Ogawahigashi, Kodaira, Tokyo 187-8502, Japan; 4 Department of Psychiatry, National Center Hospital, National Center of Neurology and Psychiatry, 4-1-1 Ogawahigashi, Kodaira, Tokyo 187-8502, Japan

## Abstract

**Introduction:**

TMEM106B is a transmembrane glycoprotein of unknown function located within endosome/lysosome compartments expressed ubiquitously in various cell types. Previously, the genome-wide association study (GWAS) identified a significant association of *TMEM106B* single nucleotide polymorphisms (SNPs) with development of frontotemporal lobar degeneration with ubiquitinated TAR DNA-binding protein-43 (TDP-43)-positive inclusions (FTLD-TDP), particularly in the patients exhibiting the progranulin (PGRN) gene (*GRN*) mutations. Recent studies indicate that TMEM106B plays a pathological role in various neurodegenerative diseases, including Alzheimer’s disease (AD). However, at present, the precise levels of TMEM106B expression in AD brains remain unknown.

**Methods:**

By quantitative reverse transcription (RT)-PCR (qPCR), western blot and immunohistochemistry, we studied TMEM106B and PGRN expression levels in a series of AD and control brains, including amyotrophic lateral sclerosis, Parkinson’s disease, multiple system atrophy and non-neurological cases.

**Results:**

In AD brains, TMEM106B mRNA and protein levels were significantly reduced, whereas PGRN mRNA levels were elevated, compared with the levels in non-AD brains. In all brains, TMEM106B was expressed in the majority of cortical neurons, hippocampal neurons, and some populations of oligodendrocytes, reactive astrocytes and microglia with the location in the cytoplasm. In AD brains, surviving neurons expressed intense TMEM106B immunoreactivity, while senile plaques, neurofibrillary tangles and the perivascular neuropil, almost devoid of TMEM106B, intensely expressed PGRN.

**Conclusions:**

We found an inverse relationship between TMEM106B (downregulation) and PGRN (upregulation) expression levels in AD brains, suggesting a key role of TMEM106B in the pathological processes of AD.

## Introduction

Frontotemporal lobar degeneration (FTLD) provides the second most common cause of presenile dementia worldwide. The first international genome-wide association study of FTLD with ubiquitinated TAR DNA-binding protein-43-positive inclusions (FTLD-TDP) identified a significant association with three distinct single nucleotide polymorphisms (SNPs) numbered rs1020004, rs6966915, and rs1990622 (top SNP) in the transmembrane protein 106B (*TMEM106B*) gene on chromosome 7p21.3 [1]. The study also found that TMEM106B mRNA levels are elevated by greater than 2.5-fold in the frontal cortex of FTLD-TDP patients, compared with the levels of normal subjects. The minor C allele on rs1990622 in the *TMEM106B* gene confers significant protection against development of FTLD, most notably in the patients with the progranulin (PGRN) gene (*GRN*) mutations [1]. This association is replicated in independent cohorts [2,3]. A number of previous studies showed that all *GRN* mutations cause FTLD-TDP by the mechanism of haploinsufficiency due to nonsense-mediated decay of mutated mRNAs [4,5]. A different study validated a substantial increase in TMEM106B mRNA and protein levels in FLTD-TDP brains with *GRN* mutations [6].

TMEM106B is a type II transmembrane glycoprotein of unknown function located within the late endosome/lysosome compartments expressed ubiquitously in various cell types, where the levels of TMEM106B expression are regulated by lysosomal activities [7,8]. In rat neurons in culture, TMEM106B plays a pivotal role in dendritic trafficking of lysosomes [9]. PGRN is a secreted glycoprotein with pleitropic functions involved in embryogenesis, oncogenesis, and inflammation, widely expressed in epithelial cells of the skin, gastrointestinal tract and the reproductive system, leukocytes, and neurons in the central nervous system [10,11]. Sortilin, serving as a cell-surface receptor for PGRN, regulates trafficking and targeting of PGRN to lysosomes [12]. The risk T allele on rs1990622 in the *TMEM106B* gene is linked to low plasma PGRN levels, suggesting that *TMEM106B* SNPs modulate secreted levels of PGRN [13,14]. A nonsynonymous SNP numbered rs3173615 (p.T185S) located in exon 6 of the *TMEM106B* gene shows complete linkage disequilibrium with rs1990622 [3,13,15]. The expression levels of the protective isoform S185 are always lower than those of the risk isoform T185, attributable to accelerated degradation of the S185 protein, suggesting that increased expression of the T185 protein might perturb the endolysosomal pathway [3]. Actually, overexpression of TMEM106B induces enlargement of lysosomes and inhibits lysosomal degradation of PGRN [8]. Importantly, the frequency of carriers homozygous for S185 on rs3173615 is reduced in the patients with C9orf72 repeat expansions, the most common genetic cause for FTLD [15], whereas the risk T allele on rs1990622 is positively associated with later age at onset and death in C9orf72 repeat expansion carriers [16].

A recent study showed that TMEM106B genotypes influence the development of cognitive impairment in amyotrophic lateral sclerosis (ALS) patients [17]. The risk T allele on rs1990622 in the *TMEM106B* gene is significantly associated with poor cognitive performance in ALS patients. Furthermore, the frequency of the protective C allele on rs1990622 is reduced in Alzheimer’s disease (AD) cases presenting with TDP-43 pathology [18]. The interplay between TMEM106B and APOE genotypes increases AD risk in a Han Chinese population [19]. All of these observations suggest that TMEM106B plays a key role in the pathology not only of FTLD-TDP but also of other neurodegenerative diseases, such as AD. The precise levels of TMEM106B expression in AD brains, however, remain unknown at present. In the present study, we characterized TMEM106B and PGRN expression levels in AD and non-AD brains by quantitative reverse transcriptase-polymerase chain reaction (qPCR), western blot and immunohistochemistry. We found that the levels of TMEM106B expression are substantially reduced, while those of PRGN are elevated in AD brains.

## Materials and methods

### Human brain tissues

The serial sections of the frontal cortex and the hippocampus were prepared from autopsied brains of six sporadic AD patients, composed of three men and three women with a mean age of 73 ± 9 years, and 13 non-AD patients, composed of six men and seven women with a mean age of 74 ± 8 years, as described previously [20]. The non-AD group includes four normal subjects that died of non-neurological causes (NC), three patients with sporadic Parkinson’s disease (PD), four patients with sporadic ALS, and two patients with sporadic multiple system atrophy (MSA). The demographic profile of the cases examined is presented in Table 1. All AD cases met with the Consortium to Establish a Registry for Alzheimer’s Disease criteria for diagnosis of definite AD [21]. They were categorized into stage C of amyloid deposition and into stage VI of neurofibrillary degeneration, following the Braak staging system [22]. Autopsies on all subjects were performed at the National Center Hospital, National Center of Neurology and Psychiatry, Japan or the Kohnodai Hospital, National Center for Global Health and Medicine, Japan. In all cases, written informed consent was obtained. The Ethics Committee of the National Center of Neurology and Psychiatry for the Human Brain Research, the Ethics Committee of the National Center for Global Health and Medicine on the Research Use of Human Samples, and the Human Research Ethics Committee of the Meiji Pharmaceutical University approved the present study.

**Table 1:** Demographic profile of the cases examined in the present study

**Case number**	**IHC**	**qPCR/WB**	**Cause of death**	**Brain weight (grams)**	**Postmortem interval (hours)**	**Braak staging (amyloid deposition/neurofibrillary degeneration)**	**pTDP-43 immunoreactivity**		**p.T185S genotype**
							**Frontal cortex**	**Hippocampus**	
NC1	+	+	Acute myocardial infarction	1,130	1.4	A/II	–	–	T/T
NC2	+	+	Acute myocardial infarction	1,350	1.6	0/II	–	–	T/S
NC3	+	+	Lung cancer	1,060	3.9	A/II	–	–	T/S
NC4	+	+	Dissecting aortic aneurysm	1,400	4.8	A/I	–	–	T/T
AD1	+	+	Pneumonia	1,000	1.1	C/VI	+	+	T/S
AD2		+	Pneumonia	1,230	14	C/VI			T/S
AD3	+	+	Pneumonia	1,220	10.5	C/VI	–	+	T/S
AD4	+	+	Pneumonia	1,240	8.1	C/VI	–	–	T/S
AD5	+	+	Lung cancer	1,090	4.5	C/VI	–	–	T/T
AD6	+	+	Pulmonary infarction	840	3	C/VI	–	+	T/S
AD7		+	Respiratory failure by aspiration	1,200	3.8	B/IV			T/S
AD8	+		Pneumonia	1,060	8	C/VI	–	+	
PD1		+	Pneumonia	1,330	9.5	B/IV			S/S
PD2	+	+	Pneumonia	1,130	2.5	B/II	–	–	T/S
PD3	+	+	Respiratory failure by aspiration	910	2.5	B/II	–	–	T/S
PD4		+	Colon cancer	1,430	4	A/I			S/S
PD5	+		Pneumonia	1,320	9.3	C/III	–	–	
ALS1	+	+	Respiratory failure	1,480	10.5	0/0	–	–	T/S
ALS2	+	+	Respiratory failure	1,090	1.3	0/I	+	+	T/T
ALS3	+	+	Respiratory failure	1,560	3	0/I	+	+	T/S
ALS4	+	+	Respiratory failure	1,320	10	0/II	+	–	S/S
ALS5		+	Respiratory failure	1,360	2.5	B/I			T/S
ALS6		+	Respiratory failure	1,600	13	B/I			T/S
MSA1	+		Pneumonia, septicemia	1,040	1.5	0/I	–	–	
MSA2	+		Pneumonia	1,090	12	A/I	–	–	

The demographic profile of the cases processed for immunohistochemistry (IHC), quantitative reverse transcriptase-polymerase chain reaction (qPCR), and western blotting (WB) is shown with the case number, the cause of death, brain weight, the postmortem interval, the Braak staging (amyloid deposition/neurofibrillary degeneration), phosphorylated TAR DNA-binding protein-43 (pTDP-43) immunoreactivity in the frontal cortex and the hippocampus, and the p.T185S genotype of the rs3173615 single nucleotide polymorphism. AD, Alzheimer’s disease; ALS, amyotrophic lateral sclerosis; MSA, multiple system atrophy; NC, non-neurological causes; PD, Parkinson’s disease.

### Immunohistochemistry

The brain tissues were fixed with 4% paraformaldehyde and embedded in paraffin. After deparaffination, tissue sections were heat-treated in 10 mM citrate sodium buffer, pH 6.0, by autoclaving at 110°C for 15 minutes in a temperature-controlled pressure chamber (Biocare Medical, Concord, CA, USA). The sections were processed for immunohistochemistry, according to the methods described previously [23]. In brief, the tissue sections were incubated at room temperature for 15 minutes with 3% hydrogen peroxide-containing methanol to block the endogenous peroxidase activity, and were also incubated with phosphate-buffered saline containing 10% normal goat or rabbit serum at room temperature for 15 minutes to block nonspecific staining. The sections were then incubated at 4°C overnight with a rabbit polyclonal anti-TMEM106B antibody raised against a peptide spanning amino acid residues 1 to 50 of the human TMEM106B protein at a concentration of 0.1 μg/ml (A303-439A; Bethyl Laboratories, Montgomery, TX, USA), a rabbit monoclonal anti-PGRN antibody raised against a synthetic peptide corresponding to the residues in the human PGRN protein at a dilution of 1:1,000 (EPR3781; Abcam, Cambridge, UK), or a mouse monoclonal anti-pS409/410 TDP-43 antibody raised against a phosphopeptide composed of CMDSKpSpSGWGM at a dilution of 1:500 (TIP-PTD-M01; Cosmo Bio, Tokyo, Japan). The specificity of A303-439A and EPR3781 antibodies was validated individually by western blot analysis of the corresponding recombinant proteins expressed in human cell lines in culture. After washing with phosphate-buffered saline, the tissue sections were labeled at room temperature for 30 minutes with peroxidase-conjugated secondary antibodies (Nichirei, Tokyo, Japan), followed by incubation with diaminobenzidine tetrahydrochloride substrate (Vector, Burlingame, CA, USA). The sections were processed for a counterstain with hematoxylin. For negative controls, the primary antibody was omitted from the reaction.

### Reverse transcriptase-polymerase chain reaction analysis

The source of human neural cell lines processed for reverse transcriptase-polymerase chain reaction (PCR) was described elsewhere. Total cellular RNA was extracted using TRIZOL (Invitrogen, Carlsbad, CA, USA). RNA treated with DNase I was processed for cDNA synthesis using oligo(dT)_20_ primers and SuperScript II reverse transcriptase (Invitrogen). cDNA was then amplified by PCR using HotStar Taq DNA polymerase (Qiagen, Valencia, CA, USA) and a panel of sense and antisense primer sets as follows: 5′-ctgacctgttcatacctgagccat-3′ and 5′-tgggagatatagaccagggttgca-3′ for a 168 base pair (bp) product of the human *TMEM106A* gene (NCBI Reference Sequence Number NM_145041); 5′-aggaagaattcctagggggcaaga-3′ and 5′-atttcacgtcgatagagcgaggga-3′ for a 173 bp product of the human *TMEM106B* gene (NM_018374); 5′-cgtgatttcccacagttccatgag-3′ and 5′-aagtacgtgatcttcagccagtcc-3′ for a 115 bp product of the 3′ noncoding region of the human *TMEM106B* gene (NM_018374); 5′-atacattggcctcatgacccagag-3′ and 5′-cttgggaacatatgctgtgctctc-3′ for a 140 bp product of the human *TMEM106C* gene (NM_024056); 5′-tgagggacagtactgaagactctg-3′ and 5′-tctgacagggaaggccttagattg-3′ for a 167 bp product of the human *GRN* gene (NM_002087); 5′-atgaggaggaaggagagaagggga-3′ and 5′-ccttccctttcctgtctgagtctc-3′ for a 188 bp product of the human glial fibrillary acidic protein (*GFAP*) gene (NM_002055); 5′-gagaaaggaacatccggaacagcc-3′ and 5′-tgggagtgccctctcttgctaaca-3′ for a 180 bp product of the human neurofilament, heavy polypeptide (*NFH*) gene (NM_021076); 5′-tacggagcggtcgtgtatcaggat-3′ and 5′-agctgcgtagactctgccgtaact-3′ for a 132 bp product of the human RNA binding protein, fox-1 homolog (*Caenorhabditis elegans*)-3 (*RBFOX3*, *also named NEUN*) gene (NM_001082575); and 5′-ccatgttcgtcatgggtgtgaacca-3′ and 5′-gccagtagaggcagggatgatgttc-3′ for a 251 bp product of the glyceraldehyde-3-phosphate dehydrogenase (*G3PDH*) gene (NM_002046).

For qPCR, cDNA prepared from frozen human brain tissues and a reference RNA of the human frontal cortex (AM6810; Invitrogen/Ambion) was amplified by PCR in a LightCycler ST300 (Roche Diagnostics, Tokyo, Japan) using SYBR Green I and the primer sets described above. The expression levels of target genes were standardized against the levels of G3PDH detected in the corresponding cDNA samples. All assays were performed in triplicate.

### p.T185S genotyping

The rs3173615 SNP composed of p.T185S (C760G) in exon 6 of the human *TMEM106B* gene was studied by direct sequencing of a 226 bp product amplified from brain cDNA by PCR using a primer set of 5′-cagcctatgtcagttatgatg-3′ and 5′-tctgctataacggtaggtact-3′. The representative data are shown in Figure S1a,b,c in Additional file 1.

### Vector construction

To study the specificity of anti-TMEM106B antibody, the full-length open reading frame of the human *TMEM106A* gene, the human *TMEM106B* gene, the human *TMEM106C* gene, or the human *GRN* gene was amplified by PCR using PfuTurbo DNA polymerase (Agilent Technologies, Palo Alto, CA, USA) and the set of sense and antisense primers. Subsequently, PCR products were cloned in the expression vector pcDNA4/HisMax-TOPO (Invitrogen) to express a fusion protein with an N-terminal Xpress tag. The vectors were transfected in HeLa cells, SK-N-SH cells, or HEK293 cells using Lipofectamine 2000 reagent (Invitrogen) for transient expression.

### Western blot analysis

To prepare total protein extract, cultured cells and frozen brain tissues were homogenized in RIPA buffer (Sigma, St. Louis, MO, USA), NP-40 lysis buffer (homemade), or buffer containing 8 M urea, 2% CHAPS, 0.5% carrier ampholytes pH 4 to 7, 20 mM dithiothreitol supplemented with a cocktail of protease inhibitors (Sigma) – this homogenization was then followed by centrifugation at 12,000 rpm for 10 minutes at room temperature to harvest the supernatant. The protein was separated on 12% SDS-PAGE gel. After gel electrophoresis, the protein was transferred onto nitrocellulose membranes, followed by incubation at room temperature overnight with the anti-TMEM106B antibody A303-439A, rabbit polyclonal anti-TMEM106B antibody raised against a peptide spanning amino acid residues 101 to 200 of the human TMEM106B protein (bs-11694R; Bioss, Boston, MA, USA), rabbit polyclonal anti-TMEM106B antibody raised against the human TMEM106B-GST fusion protein (20995-1-AP; Proteintech, Chicago, IL, USA), or mouse monoclonal anti-Xpress antibody (Invitrogen). The membranes were then incubated at room temperature for 30 minutes with horseradish peroxidase-conjugated anti-rabbit IgG (Santa Cruz Biotechnology, Santa Cruz, CA, USA). The specific reaction was visualized by exposing them to a chemiluminescent substrate (Pierce, Rockford, IL, USA). After the antibodies were stripped by incubating the membranes at 50°C for 30 minutes in stripping buffer, composed of 62.5 mM Tris–HCl, pH 6.7, 2% SDS, and 100 mM 2-mercaptoethanol, the membranes were processed for relabeling with anti-heat shock protein HSP60 antibody (sc-1052; Santa Cruz Biotechnology), serving as an internal control of protein loading. The signal intensity of TMEM106B-immunoreactive bands was quantified using ImageJ software (National Institute of Health, Bethesda, MD, USA), and the expression levels were standardized individually by the corresponding HSP60 signal intensity.

### Statistical analysis

The statistical significant difference between two groups was evaluated by Student’s *t* test. A significant difference among >2 groups was evaluated by one-way analysis of variance followed by Turkey’s *post hoc* test. The differences in the frequency of T185 and S185 isoforms between the groups were evaluated after allocating score 0 to the T185 allele and score 1 to the S185 allele. The correlation between two groups was evaluated by Pearson’s correlation coefficient test. *P* < 0.05 in the two-tailed test was considered significant.

## Results

### Evolutional conservation of TMEM106B

**Figure 1:**
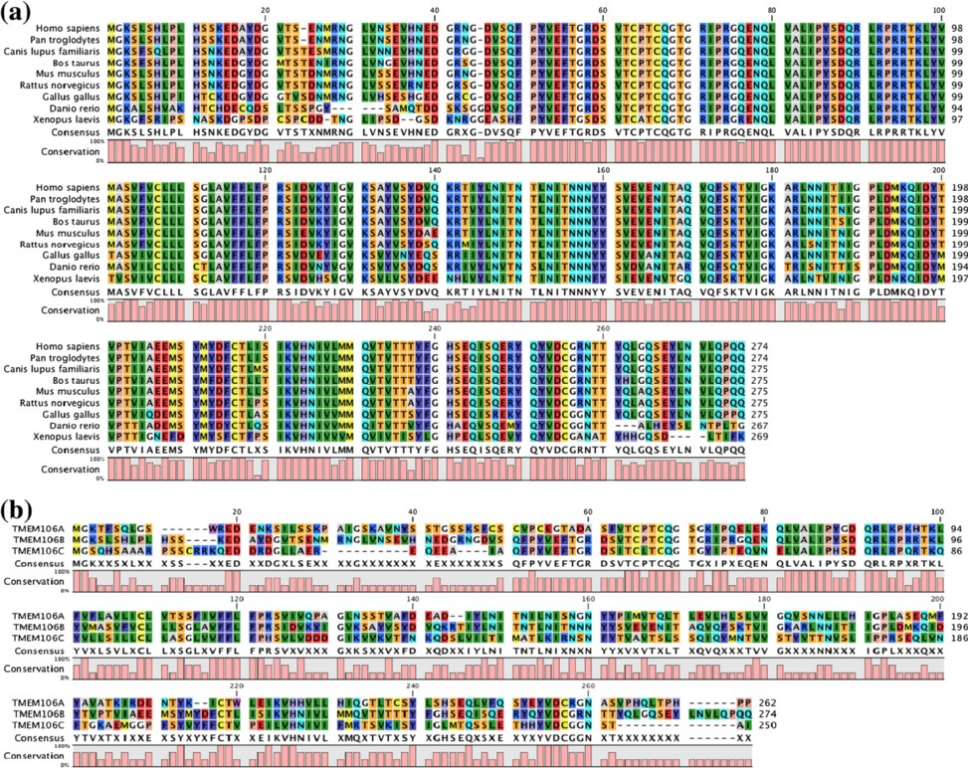
**Multiple sequence alignment of TMEM106B protein.** Multiple amino acid sequence alignment was performed by importing the corresponding amino acid sequences into CLC Free Workbench (CLC Bio/Qiagen, Aarhus, Denmark). **(a)** Multiple amino acid sequence alignment of TMEM106B orthologs derived from *Homo sapiens*, *Pan troglodytes*, *Canis lupus familiaris*, *Bos Taurus*, *Mus musclus*, *Rattus norvegicus*, *Gallus gallus*, *Danio rerio*, and *Xenopus laevis*. **(b)** Multiple amino acid sequence alignment of the human TMEM106A, TMEM106B, and TMEM106C proteins.

Multiple sequence alignment analysis revealed that the *TMEM106B* gene is highly conserved in various vertebrates through evolution. The amino acid sequence of the human TMEM106B protein was 100%, 96%, 95%, 96%, 95%, 87%, 75%, and 68% identical to the sequences of orthologs derived from *Pan troglodytes*, *Canis lupus familiaris*, *Bos Taurus*, *Mus musclus*, *Rattus norvegicus*, *Gallus gallus*, *Danio rerio*, and *Xenopus laevis*, respectively (Figure 1a). Furthermore, the amino acid sequence of the human TMEM106B protein was 49% and 47% identical to the sequences of the human TMEM106A and TMEM106C proteins, respectively (Figure 1b), suggesting that the latter two represent paralogues of TMEM106B.

### Universal expression of TMEM106A, TMEM106B, TMEM106C, and PGRN mRNAs in human neural cells

**Figure 2:**
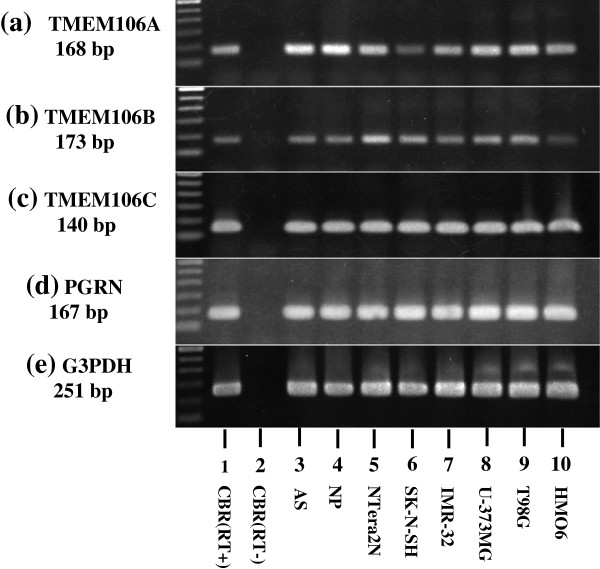
**Universal expression of TMEM106B mRNAs in human neural cells.** mRNA expression was studied by reverse transcriptase (RT)-polymerase chain reaction (PCR) in human tissues and cultured cells. **(a)** TMEM106A, **(b)** TMEM106B, **(c)** TMEM106C, **(d)** progranulin (PGRN), and **(e)** G3PDH, a housekeeping gene for a positive control. The lanes indicate (1) the frontal cortex of the human cerebrum (CBR) with inclusion of the RT step, (2) CBR without inclusion of the RT step, (3) astrocytes (AS), (4) neuronal progenitor (NP) cells, (5) NTera2 teratocarcinoma-derived neurons, (6) SK-N-SH neuroblastoma, (7) IMR-32 neuroblastoma, (8) U-373MG glioblastoma, (9) T98G glioblastoma, and (10) HMO6 microglia. TMEM106A, TMEM106B, TMEM106C, and PGRN were amplified for 35 cycles, while G3PDH was amplified for 28 cycles.

By reverse transcriptase-PCR, all cells and tissues examined – including the human cerebrum, astrocytes, neuronal progenitor cells, NTera2 teratocarcinoma-derived neurons, SK-N-SH neuroblastoma, IMR-32 neuroblastoma, U-373MG glioblastoma, T98 glioblastoma, and HMO6 immortalized microglia – expressed varying levels of TMEM106A, TMEM106B, TMEM106C, and PGRN transcripts (Figure 2a,b,c,d, lanes 1,3 to 10). The levels of G3PDH, a housekeeping gene, were almost constant in the cells and tissues examined (Figure 2e, lanes 1,3 to 10). No products were amplified when the reverse transcription step is omitted (Figure 2a,b,c,d,e, lane 2). The expression of TMEM106A, TMEM106B, TMEM106C, and PGRN mRNAs is thus universal in human neural cell lines.

### Reduced expression of TMEM106B mRNA in Alzheimer’s disease brains

We next analyzed by qPCR the levels of TMEM106B, PGRN, and G3PDH mRNAs in frozen human brain tissues derived from four NC cases, six ALS cases, four PD cases, and seven AD cases presented in Table 1. Before starting this, we investigated the p.T185S genotype of rs3173615 in the human *TMEM106B* gene, on which the T185 isoform acts as a risk factor, while the S185 isoform serves a protective factor for development of FTLD with *GRN* mutations. In the brains examined, the T185/T185 homozygote consisted of four cases (19.0%), the T185/S185 heterozygote consisted of 14 cases (66.7%), and the S185/S185 homozygote consisted of three cases (14.3%) (Table 1), consistent with the genotyping data of HapMap-JPT [24]. The frequency of T185 and S185 isoforms was thus not significantly different between AD and non-AD groups (*P* = 0.6134).

**Figure 3:**
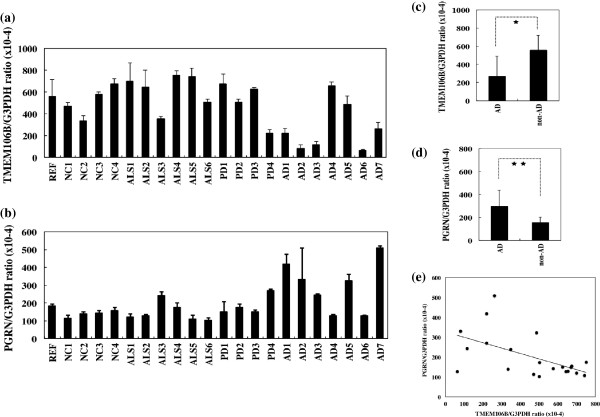
**Reduced expression of TMEM106B mRNA in Alzheimer’s disease brains.** TMEM106B and progranulin (PGRN) mRNA expression levels were studied by quantitative reverse transcriptase-polymerase chain reaction (qPCR) in human brain tissues derived from a reference of the human frontal cortex (REF), four non-neurological control cases (NC), six amyotrophic lateral sclerosis (ALS) cases, four Parkinson’s disease (PD) cases, and seven Alzheimer’s disease (AD) cases. The expression levels were standardized against those of G3PDH. **(a)** TMEM106B mRNA expression. **(b)** PGRN mRNA expression. **(c)** Difference in TMEM106B levels between AD and non-AD cases. *P = 0.0035 by Student’s *t* test. **(d)** Difference in PGRN levels between AD and non-AD cases. **P = 0.0027 by Student’s *t* test. **(e)** Pearson’s correlation between TMEM106B and PGRN mRNA levels. Pearson’s correlation coefficient indicates −0.555 (*P* = 0.0090).

**Figure 4:**
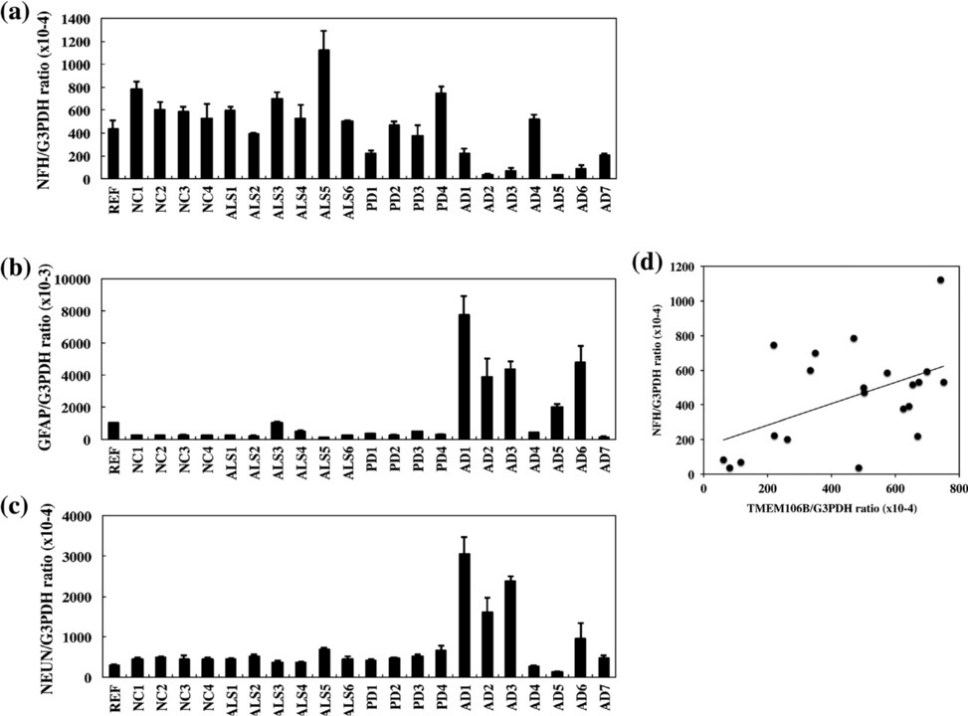
**Positive correlation between TMEM106B and neurofilament, heavy polypeptide mRNA levels.** Neurofilament, heavy polypeptide (NFH), glial fibrillary acidic protein (GFAP), and RNA-binding protein, fox-1 homolog (*Caenorhabditis elegans*)-3 (RBFOX3, NEUN) mRNA expression levels were studied by quantitative reverse transcriptase-polymerase chain reaction (qPCR) in human brain tissues derived from a reference of the human frontal cortex (REF), four non-neurological causes (NC) cases, six amyotrophic lateral sclerosis (ALS) cases, four Parkinson’s disease PD cases, and seven AD cases. The expression levels were standardized against those of G3PDH. **(a)** NFH expression. **(b)** GFAP expression. **(c)** NEUN expression. **(d)** Pearson’s correlation between TMEM106B and NFH mRNA levels. Pearson’s correlation coefficient indicates 0.496 (*P* = 0.0221).

By qPCR, AD cases showed significantly reduced mRNA levels of TMEM106B, when compared with those in non-AD cases (*P* = 0.0035) (Figure 3a,c). In contrast, AD cases showed significantly elevated mRNA levels of PGRN, with some variations among the cases, when compared with the levels in non-AD cases (*P* = 0.0027) (Figure 3b,d). Notably, a significant negative correlation was found between TMEM106B and PGRN mRNA expression levels (Pearson’s correlation coefficient = −0.555; *P* = 0.0090) (Figure 3e). Furthermore, AD cases showed significantly reduced mRNA levels of NFH and elevated mRNA levels of GFAP and NEUN, when compared with the levels in non-AD cases (*P* = 0.0003 for NFH, *P* = 0.0004 for GFAP, and *P* = 0.0156 for NEUN) (Figure 4a,b,c). Importantly, a significant positive correlation was found between TMEM106B and NFH mRNA expression levels (Pearson’s correlation coefficient = 0.496; *P* = 0.0221) (Figure 4d).

Moreover, we studied by qPCR the levels of TMEM106A and TMEM106C mRNAs in AD and non-AD brains. Both were markedly elevated in AD brains, compared with the levels in non-AD brains (*P* = 0.0002 for TMEM106A and *P* = 0.0005 for TMEM106C) (Figure S2a,b,c,d in Additional file 2). The expression of TMEM106B paralogues was uniquely regulated in the opposite direction to the expression levels of TMEM106B.

### Characterization of the specificity of anti-TMEM106B antibody

**Figure 5:**
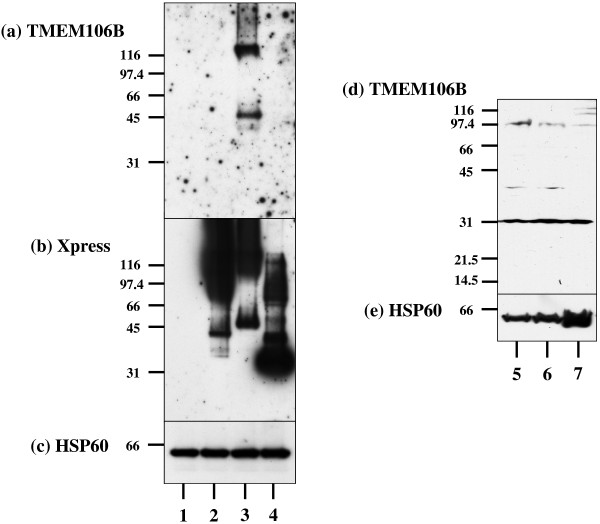
**Characterization of anti-TMEM106B antibody.** The full-length open reading frame (ORF) cloned in the vector that expresses a fusion protein with an N-terminal Xpress tag was transiently expressed in HeLa cells. Total protein extract was processed for western blot. Lanes represent the protein of (1) untransfected cells and the cells expressing (2) TMEM106A, (3) TMEM106B, or (4) TMEM106C, and the protein of (5) human brain #1, (6) human brain #2, or (7) IMR-32 neuroblastoma cells. Immunoblots of **(a, d)** TMEM106B (the A303-439A antibody), **(b)** Xpress, and **(c, e)** HSP60, an internal control for protein loading.

The specificity of anti-human TMEM106 antibody was verified by western blot of recombinant human TMEM106A, TMEM106B, and TMEM106C proteins tagged with Xpress expressed in HeLa cells. The A303-439A anti-TMEM106B antibody recognized 45 kDa monomeric and 120 kDa oligomeric forms of TMEM106B tagged with Xpress (Figure 5a,b, lane 3), whereas it did react either with TMEM106A or with TMEM106C (Figure 5a,b, lanes 2 and 4), validating the specificity of the A303-439A antibody. In contrast, both bs-11694R and 20995-1-AP anti-TMEM106B antibodies did not specifically react with the Xpress-tagged human TMEM106B protein (data not shown). We therefore selected A303-439A for western blot and immunohistochemistry analysis in the present study. This antibody specifically reacted with a major 31 kDa protein endogenously expressed in human brain tissues and IMR-32 neuroblastoma cells (Figure 5d, lanes 5 to 7).

### Reduced expression of TMEM106B protein in Alzheimer’s disease brains

**Figure 6:**
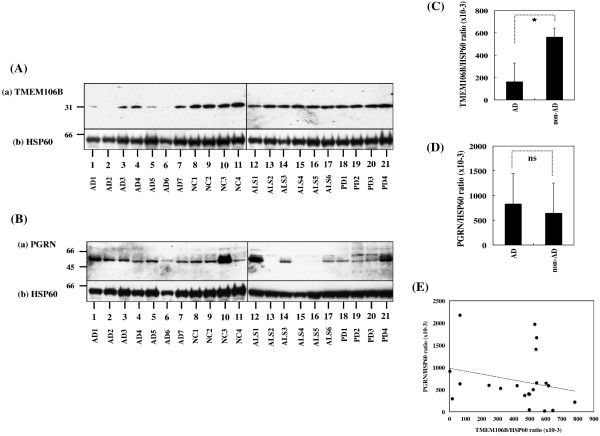
**Reduced expression of TMEM106B protein in Alzheimer’s disease brains.** Protein expression levels were studied by western blot in human brain tissues derived from four non-neurological causes (NC) cases, six amyotrophic lateral sclerosis (ALS) cases, four Parkinson’s disease (PD) cases, and seven Alzheimer’s disease (AD) cases. The expression levels were standardized against those of HSP60. **(A)** TMEM106B expression: **(a)** TMEM106B and **(b)** HSP60. **(B)** Progranulin (PGRN) expression: **(a)** PGRN and **(b)** HSP60. **(C)** Difference in TMEM106B levels between AD and non-AD cases. *P = 0.0000004 by Student’s *t* test. **(D)** Difference in PGRN levels between AD and non-AD cases. ns, non-significant (*P* = 0.5304 by Student’s *t* test). **(E)** Pearson’s correlation between TMEM106B and PGRN protein levels. Pearson’s correlation coefficient indicates −0.242 (*P* = 0.2912).

Next, we quantitatively analyzed the levels of TMEM106B, PGRN, and HSP60 proteins in frozen human brain tissues derived from four NC cases, six ALS cases, four PD cases, and seven AD cases, presented in Table 1, by western blot using the A303-439A antibody. AD cases showed significantly reduced levels of TMEM106B, when compared with the levels in non-AD cases (*P* = 0.0000004) (Figure 6Aa,C). AD cases showed a trend for elevated expression levels of PRGN when compared with the levels in non-AD cases, but the difference did not reach statistical significance (*P* = 0.5304) (Figure 6Ba,D). We found no discernible correlation between TMEM106B and PRGN protein expression levels (Pearson’s correlation coefficient = −0.242; *P* = 0.2912) (Figure 6E).

### Immunohistochemical analysis of TMEM106B expression in Alzheimer’s disease and non-Alzheimer’s disease brains

**Figure 7:**
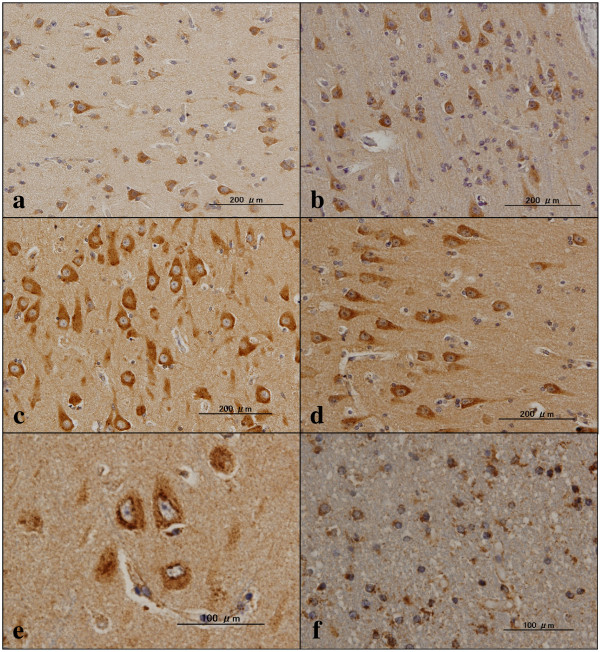
**TMEM106B immunoreactivity in non-Alzheimer’s disease brains.** Expression of TMEM106 immunoreactivity was studied in 13 non-Alzheimer’s disease brains presented in Table 1 by immunohistochemistry using the A303-439A antibody. **(a)** Non-neurological causes (NC), the frontal cortex, cytoplasmic staining of cortical neurons; **(b)** amyotrophic lateral sclerosis (ALS), the frontal cortex, cytoplasmic staining of cortical neurons; **(c)** NC, the hippocampal CA1 region, cytoplasmic staining of pyramidal neurons; **(d)** ALS, the hippocampal CA1 region, cytoplasmic staining of pyramidal neurons; **(e)** NC, the hippocampal CA1 region, intense staining of small nodular structures accumulated in the perinuclear region of pyramidal neurons; **(f)** NC, the frontal white matter, cytoplasmic staining of oligodendrocytes, reactive astrocytes, and microglia.

We next studied the expression of TMEM106B in the frontal cortex and the hippocampus of six AD cases and 13 non-AD cases, composed of four NC cases, four ALS cases, three PD cases, and two MSA cases, presented in Table 1, by immunohistochemistry using the A303-439A antibody. Before starting this, we investigated TDP-43 pathology in the brains examined. Among 19 cases, four AD cases and three ALS cases – but no cases of NC, PD, or MSA – showed neuronal or glial pTDP-43 immunoreactivity in the frontal cortex and/or the hippocampus (Table 1; Figure S3a,b,c,d in Additional file 3). In all cases examined, TMEM106B was expressed in the majority of cortical neurons, hippocampal pyramidal neurons and dentate gyrus granule cells, located in the cytoplasm by forming fine granular structures, particularly enriched in the soma and in proximal neurites (Figure 7a,b,c,d). TMEM106B immunoreactivity was occasionally concentrated in the perinuclear region by forming small nodular structures in some populations of hippocampal pyramidal neurons (Figure 7e). Furthermore, subpopulations of oligodendrocytes, reactive astrocytes, and microglia expressed TMEM106B intensely, located in the cytoplasm (Figure 7f; Figure S4b,c in Additional file 4). Neuronal cytoplasmic TMEM106B immunoreactivity was greatly reduced after absorption of the antibody by extract of the Xpress-tagged TMEM106B protein (Figure S4e,f in Additional file 4).

**Figure 8:**
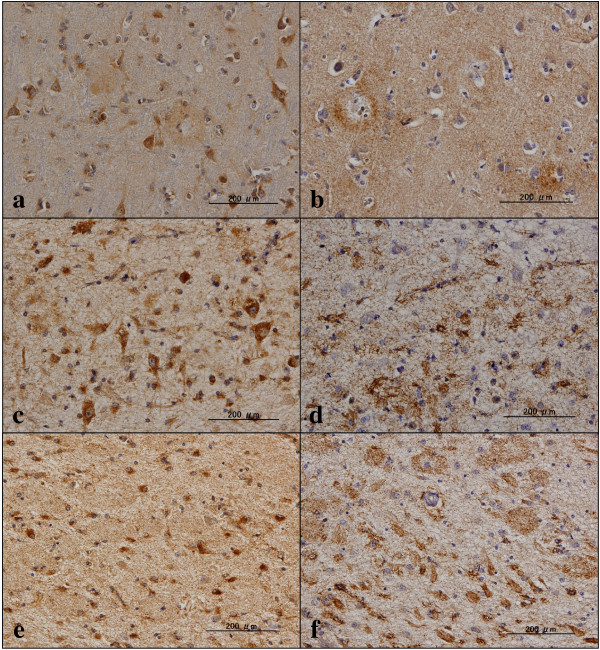
**TMEM106B and PGRN immunoreactivities in Alzheimer’s disease brains.** Expression of TMEM106 and progranulin (PGRN) immunoreactivities was studied in six Alzheimer’s disease brains presented in Table 1 by immunohistochemistry using the A303-439A antibody. **(a)** TMEM106B, the frontal cortex, moderate neuronal cytoplasmic staining and faint senile plaque staining; **(b)** PGRN, same region as **(a)**, moderate senile plaque staining and diffuse neuropil staining; **(c)** TMEM106B, the hippocampal CA1 region, intense neuronal and astroglial cytoplasmic staining; **(d)** PGRN, same region as **(c)**, intense perivascular neuropil staining; **(e)** TMEM106B, the hippocampal CA1 region, no staining of senile plaques and neurofibrillary tangles; **(f)** PGRN, same region as **(e)**, moderate staining of numerous senile plaques and neurofibrillary tangles.

In AD brains, cortical neurons and hippocampal pyramidal neurons were greatly reduced in number, along with substantial reduction of TMEM106B-expressing neurons. However, surviving neurons in AD brains moderately or intensely expressed TMEM106B immunoreactivity in the cytoplasm (Figure 8a,c). Senile plaques were most often unlabeled and rarely faintly labeled by the A303-439A antibody (Figure 8a,e). In contrast, senile plaques, neurofibrillary tangles, and the perivascular neuropil were frequently and intensely labeled with anti-PGRN antibody EPR3781 (Figure 8b,d,f). Some populations of activated microglia also expressed PGRN (Figure S4d in Additional file 4). The vacuoles of granulovacuolar degeneration (GVD) often found in pyramidal neurons of the hippocampal CA1 region were devoid of TMEM106B immunoreactivity (Figure S4a in Additional file 4).

### Overexpression of TMEM106B and PGRN did not alter their mRNA expression levels

Finally, we studied by qPCR the direct inverse relationship between TMEM106B and PGRN mRNA expression in SK-N-SH neuroblastoma cells following overexpression of Xpress-tagged TMEM106B, PGRN, and LacZ proteins (Figure S5a, lanes 2 to 4 in Additional file 5). Transient overexpression of the TMEM106B, PGRN, or LacZ transgene did not significantly alter the levels of endogenous TMEM106B and PGRN mRNAs (*P* = 0.4726 for TMEM106B and *P* = 0.1204 for PGRN) (Figure S5c,d in Additional file 5). These results suggest that TMEM106B is not directly involved in transcriptional regulation of the *GRN* gene, and *vice versa*.

## Discussion

By multiple sequence alignment analysis, we found that the *TMEM106B* gene is highly conserved in various vertebrates through evolution, and it shows substantial homology to both *TMEM106A* and *TMEM106C* genes that represent *TMEM106B* paralogues. Recent studies indicate that TMEM106B plays a pathological role in a wide range of neurodegenerative diseases [17-19,25]. By qPCR, western blot and immunohistochemistry, we studied TMEM106B and PGRN expression levels in a series of AD and non-AD brains. We found that TMEM106B mRNA and protein levels are significantly reduced in AD brains, while PGRN mRNA levels were elevated in AD brains, compared with the levels in non-AD brains. In all brains examined, TMEM106B was expressed in the majority of cortical neurons, hippocampal neurons, and subpopulations of oligodendrocytes, reactive astrocytes, and microglia. These observations largely agree with a recent report showing widespread expression of TMEM106B in normal human brains [26]. Although cortical neurons were most evidently lost in AD brains at advanced stages compared with non-AD brains, surviving neurons expressed fairly intense TMEM106B immunoreactivity, suggesting the possibility that reduced expression of TMEM106B in AD brains might simply reflect greater loss of neurons in the cerebral cortex. In contrast, senile plaques, neurofibrillary tangles, and the perivascular neuropil expressed intense PGRN immunoreactivity. These observations are well consistent with previous studies showing enhanced expression of PGRN in microglia, neurons, and neurites surrounding amyloid plaques in AD brains [4,10,27]. Importantly, we found that AD cases show significantly reduced mRNA levels of NFH and elevated mRNA levels of GFAP, when compared with the levels in non-AD cases, reflecting enhanced neuronal loss and astrogliosis in AD brains. Furthermore, we identified a discernible positive correlation between TMEM106B and NFH mRNA expression levels. Unexpectedly, we found a significant elevation in NEUN mRNA levels, a nuclear marker specific for subpopulations of neurons, in AD brains.

The rs1990622 SNP in the *TMEM106B* gene, being in complete linkage disequilibrium with the coding rs3173615 SNP of p.T185S, is closely associated with FTLD-TDP in the patients with *GRN* mutations, who are characterized by lower plasma PGRN levels [3,25]. Previous studies also showed that TMEM106B mRNA and protein levels are elevated in FLTD-TDP brains with *GRN* mutations [1,6]. The expression levels of the risk isoform T185 are much higher than those of the protective isoform S185 owing to destabilization of the S185 protein [3]. Overexpression of TMEM106B inhibits lysosomal function, thereby leading to disturbed turnover of PGRN [8]. An inverse relationship has thus been established in the expression levels between TMEM106B (upregulation) and PGRN (downregulation) in FTLD-TDP. PGRN acts as a pivotal neuronal survival factor, potentially deficient in the brains of neurodegenerative diseases [10,11]. All of these observations suggest that deficient expression of PGRN triggered by elevated expression of TMEM106B promotes neurodegeneration. However, in contrast to FLTD-TDP brains with *GRN* mutations, we found that TMEM106B mRNA and protein levels are reduced in AD brains. In the present study, the frequency of T185 and S185 isoforms was not significantly different between AD and non-AD cases. As a result, we unexpectedly found a reverse inverse correlation between TMEM106B (downregulation) and PGRN (upregulation) in AD brains at least at mRNA expression levels. The possible scenario that TMEM106B plays a protective role against the neurodegenerative processes in AD could therefore be raised, although further studies on *in vitro* and *in vivo* TEME106B knockdown models are required to evaluate this possibility.

In the present study, AD cases showed significantly elevated mRNA levels of PGRN, when compared with the levels in non-AD cases. However, we did not find a significant elevation of PGRN protein levels in AD brains, leading to no obvious inverse correlation between TMEM106B and PGRN protein expression levels. The inconsistency between PGRN mRNA and protein levels is attributable to the complex post-transcriptional modification of the PGRN protein. The PGRN protein contains 7.5 tandem repeats of 12 cysteinyl motifs separated by linkers. When secreted extracellularly, PGRN – cleaved by elastase and matrix metalloproteases within linker regions – generates several smaller fragments called granulins (GRNs), composed of GRN A to G and paragranulin or epithelins.

Importantly, the full-length PRGN and its cleaved fragment GRNs play a discrete role in regulation of various biological responses [10,11]. PGRN exhibits neurotrophic and anti-inflammatory activities, whereas GRNs serve as a proinflammatory mediator. Expression levels of PGRN, whose release is facilitated by anti-inflammatory stimuli in microglia, are elevated in multiple sclerosis brains [28,29]. Astrocytes produce large amounts of secretory leukocyte protease inhibitor, a negative regulator of proteolytic processing of PGRN, in response to proinflammatory stimuli [28]. In contrast, PGRN acts as a chemotactic factor for microglia capable of producing large amounts of reactive oxygen species, although microglia, following exposure to PGRN, show an enhanced capacity to phagocytose amyloid-β_1-42_[30]. At present, therefore, whether upregulated expression of PGRN in AD brains plays a neuroprotective or neurotoxic role remains unknown. We found that overexpression of either TMEM106B or PGRN transgene in SK-N-SH neuroblastoma cells does not immediately affect endogenous levels of PGRN or TMEM106B mRNA, excluding the direct interaction between both in transcription regulation of mutual genes. It is worthy of note that TMEM106B is co-localized with PGRN within the endosome/lysosome compartments [3], and treatment with inhibitors of lysosomal acidification, such as bafilomycin A1, ammonium chloride, and chloroquine, elevates TMEM106B levels in mouse neural cells [8].

A recent study found that the frequency of the protective C allele on rs1990622 in the *TMEM106B* gene, showing the complete linkage disequilibrium with p.T185S on rs3173615 [3,13,15], is reduced in AD cases exhibiting TDP-43 pathology [18]. In contrast, we found no difference in the frequency of T185 and S185 isoforms on rs3173615 between AD and non-AD cases. TDP-43, a nuclear RNA/DNA-binding protein capable of interacting with UG/TG repeat stretches of target RNAs/DNAs, plays a key role in regulation of transcription, alternative splicing, mRNA stability and transport, and microRNA biogenesis, actively involved in the pathogenesis of FTLD/ALS termed TDP-43 proteinopathy [31]. Because TMEM106B is identified as a direct target for TDP-43-regulated gene expression [32], the cytoplasmic sequestration of TDP-43 in TDP-43 proteinopathy might induce deregulated expression of TMEM106B in neurons containing TDP-43-positive inclusions. In the present study, four out of six AD cases showed TDP-43 pathology in the frontal cortex and/or the hippocampus. Among these we found that three cases (AD1, AD3, and AD6) show markedly reduced TMEM106B mRNA and protein expression levels (see Figures 3a, 6A), suggesting an involvement of aberrant regulation of the *TMEM106B* gene by TDP-43 in the pathogenesis of AD, although larger cohorts are required to evaluate this possibility.

In contrast to downregulation of TMEM106B expression, the expression of two paralogues of TMEM106B (TMEM106A and TMEM106C) was markedly upregulated at mRNA levels, almost specifically expressed in AD brains. The corresponding genes are located in different chromosomes – that is, TMEM106A (17q21.31), TMEM106B (7p21.3), and TMEM106C (12q13.1) – whose expression is presumably regulated by distinct mechanisms. The possibility exists that upregulation of the functionally relevant paralogues reflects a compensation for a deficiency of TMEM106B in AD brains. Further studies are required to evaluate this possibility.

In AD brains, granulovaculoar degeneration bodies, a kind of autophagosome, express immunoreactivity for charged multivesicular body protein 2B (CHMP2B), whose genetic mutations definitely cause FTLD [33]. We found that granulovacuolar degeneration vacuoles (GVD) located in hippocampal CA1 pyramidal neurons of AD brains are devoid of TMEM106B immunoreacitivity. At present, the mechanisms responsible for reduced expression of TMEM106B in AD brains remain unknown. If downregulation of TMEM106B is directly or indirectly involved in neurodegeneration, we could propose the hypothesis that TMEM106B plays a protective role against the neurodegenerative processes in AD. Worthy of note is that by analyzing the promoter region of the *TMEM106B* gene with bioinformatics tools for the Database of Transcriptional Start Site [34] and the Matrix Search for Transcription Factor Binding Sites [35], we identified three potential binding sites for POU class 2 homeobox 1 (POU2F1; OCT1), a transcription factor of the POU transcription factor family (unpublished observations) whose SNP is closely associated with the genetic risk of AD [36].

## Conclusions

TMEM106B mRNA and protein expression levels were reduced, while PGRN mRNA levels were elevated, in AD brains compared with the levels in non-AD brains. TMEM106B was expressed in the cytoplasm of cortical neurons, hippocampal neurons, and subpopulations of oligodendrocytes, reactive astrocytes, and microglia. In AD brains, surviving neurons expressed moderate/intense TMEM106B immunoreactivity, while senile plaques, neurofibrillary tangles, and the perivascular neuropil intensely expressed PGRN. These observations suggest an active role of TMEM106B in the pathological processes of AD.

## Abbreviations

AD: Alzheimer’s disease; ALS: amyotrophic lateral sclerosis; bp: base pair; FTLD: frontotemporal lobar dementia; GFAP: glial fibrillary acidic protein; GRN: granulin; NC: non-neurological causes; NEUN: RNA binding protein, fox-1 homolog (*Caenorhabditis elegans*)-3 (RBFOX3); NFH: neurofilament, heavy polypeptide; PCR: polymerase chain reaction; PD: Parkinson’s disease; PGRN: progranulin; qPCR: quantitative reverse transcriptase-polymerase chain reaction; SNP: single nucleotide polymorphism; TDP-43: TAR DNA-binding protein-43; TMEM106B: transmembrane protein 106B.

## Competing interests

The authors declare that they have no competing interests.

## Authors’ contributions

JS and KA designed the study. JS, YK, NK, and YY carried out qPCR, western blot, immunohistochemistry, and genetic analysis. TI, YS, and KA validated the pathological diagnosis of autopsied brains. JS, TI, YS, and KA cooperatively analyzed immunohistochemical data. JS drafted the manuscript. YK, NK, YY, TI, YS, and KA read the draft, critically revised the entire contents, and approved the final manuscript. All authors read and approved the final manuscript.

## Supplementary Material

Additional file 1: Figure S1 Showing p.T185S genotyping analysis. The rs3173615 SNP composed of p.T185S (C760G) in exon 6 of the human *TMEM106B* gene was studied by direct sequencing of PCR product amplified from brain cDNA. (a) T185/T185 homozygote, (b) T185/S185 heterozygote, and (c) S185/S185 homozygote. Click here for file

Additional file 2: Figure S2 Showing elevated expression of TMEM106A and TMEM106C mRNA in AD brains. The TMEM106A and TMEM106C mRNA expression levels were studied by qPCR in human brain tissues derived from a REF, four NC cases, six ALS cases, four PD cases, and seven AD cases. The expression levels were standardized against those of G3PDH. (a) TMEM106A. (b) TMEM106C. (c) Difference in TMEM106A levels between AD and non-AD cases. *P = 0.0002 by Student’s *t* test. (d) Difference in TMEM106C levels between AD and non-AD cases. **P = 0.0005 by Student’s *t* test. Click here for file

Additional file 3: Figure S3 Showing pTDP-43 immunoreactivity in AD and non-AD brains. The expression of phosphorylated TDP-43 (pTDP-43) immunoreactivity was studied in six AD brains and 13 non-AD brains presented in Table 1 by immunohistochemistry using anti-pS409/410 TDP-43 antibody. (a) AD, the hippocampal granule cell layer, neuronal cytoplasmic staining; (b) ALS, the hippocampal granule cell layer, neuronal cytoplasmic staining; (c) AD, the frontal cortex, microglial cytoplasmic staining; (d) ALS, the frontal cortex, neuronal cytoplasmic staining. Click here for file

Additional file 4: Figure S4 Showing TMEM106B and PGRN immunoreactivities in AD and non-AD brains. The expression of TMEM106 and PGRN immunoreactivities was studied in six AD brains and 13 non-AD brains presented in Table 1 by immunohistochemistry using the A303-439A antibody. (a) TMEM106B, AD, the hippocampal CA1 region, vacuoles of granulovacuolar degeneration (GVD) devoid of staining; (b) TMEM106B, AD, the hippocampal molecular layer, intense astroglial cytoplasmic staining; (c) TMEM106B, AD, the periventricular white matter, intense oligodendroglial cytoplasmic staining; (d) PGRN, AD, the frontal white matter, intense microglial cytoplasmic staining; (e) TMEM106B, PD, the frontal cortex, moderate/intense neuronal cytoplasmic staining; (f) TMEM106B after absorption of the antibody, same region as (e), diminished neuronal cytoplasmic staining. Click here for file

Additional file 5: Figure S5 Showing overexpression of TMEM106B or PGRN did not alter PGRN or TMEM106B mRNA expression levels in SK-N-SH neuroblastoma cells. SK-N-SH neuroblastoma cells expressing Xpress-tagged recombinant proteins were processed for western blot and qPCR. Immunoblot of (a) Xpress and (b) HSP60, an internal control for protein loading. Lanes represent the protein of (1) untransfected cells and the cells expressing (2) TMEM106B, (3) PGRN, and (4) LacZ tagged with Xpress. mRNA expression levels of (c) TMEM106B and (d) PGRN in SK-N-SH cells exposed to Lipofectamine 2000 alone (CNT) and following expression of TMEM106B, PGRN, and LacZ proteins tagged with Xpress. (Single star indicates P = 0.1204 by one-way ANOVA, while double star indicates P = 0.4726 by one-way ANOVA). Click here for file
