# Supplementary figures and images for: TMEM106B expression is reduced in Alzheimer’s disease brains
Source: Alzheimers Res Ther. 2014 Mar 31;6(2):17. doi: 10.1186/alzrt247 (PMC4055042; doi:10.1186/alzrt247)

## Slide 1
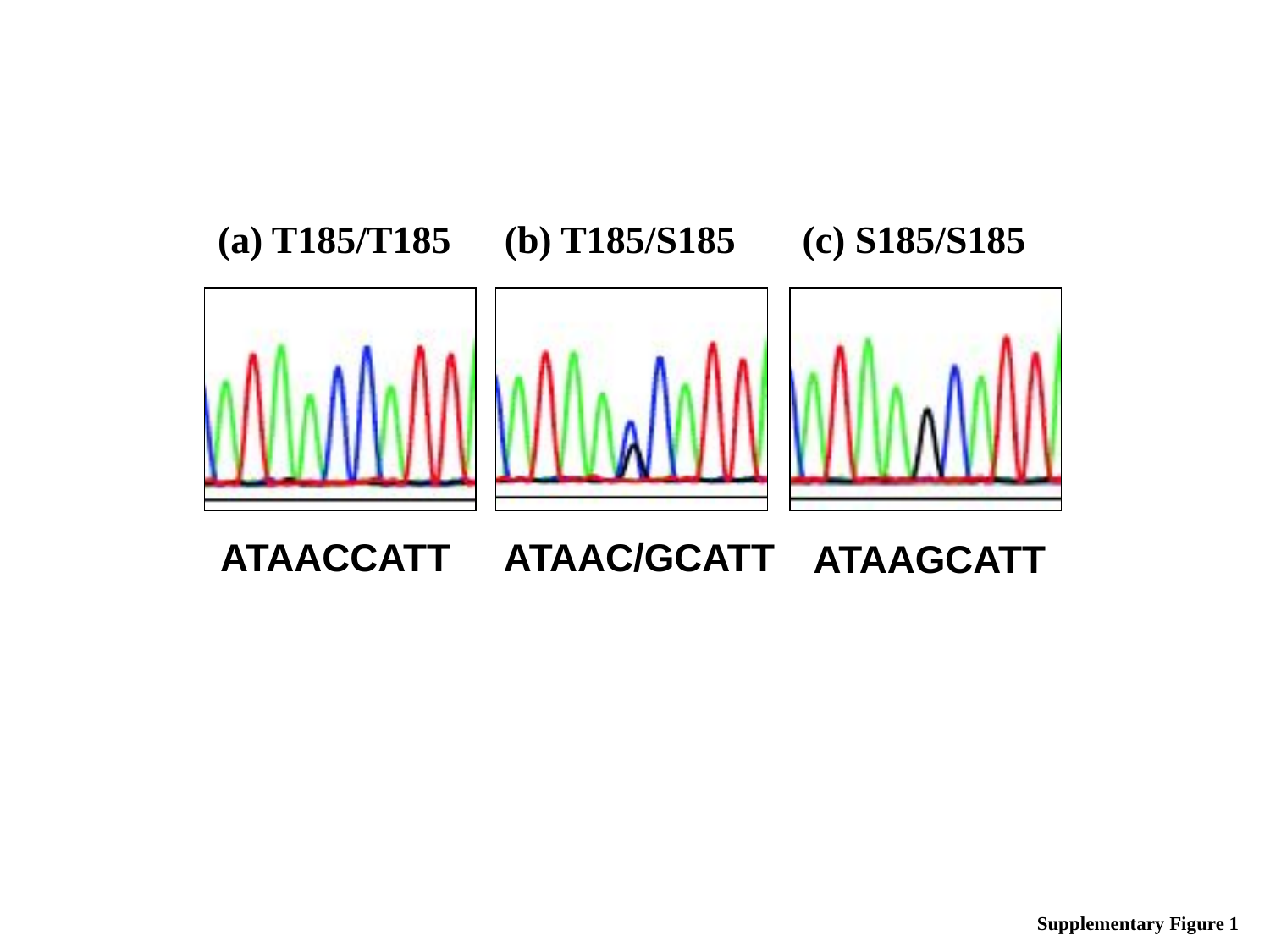

(a) T185/T185
(b) T185/S185
(c) S185/S185
ATAACCATT
ATAAC/GCATT
ATAAGCATT
Supplementary Figure 1

Supplement: Additional file 1: Figure S1 — Showing p.T185S genotyping analysis. The rs3173615 SNP composed of p.T185S (C760G) in exon 6 of the human TMEM106B gene was studied by direct sequencing of PCR product amplified from brain cDNA. (a) T185/T185 homozygote, (b) T185/S185 heterozygote, and (c) S185/S185 homozygote. [file alzrt247-S1.ppt]

## Slide 1
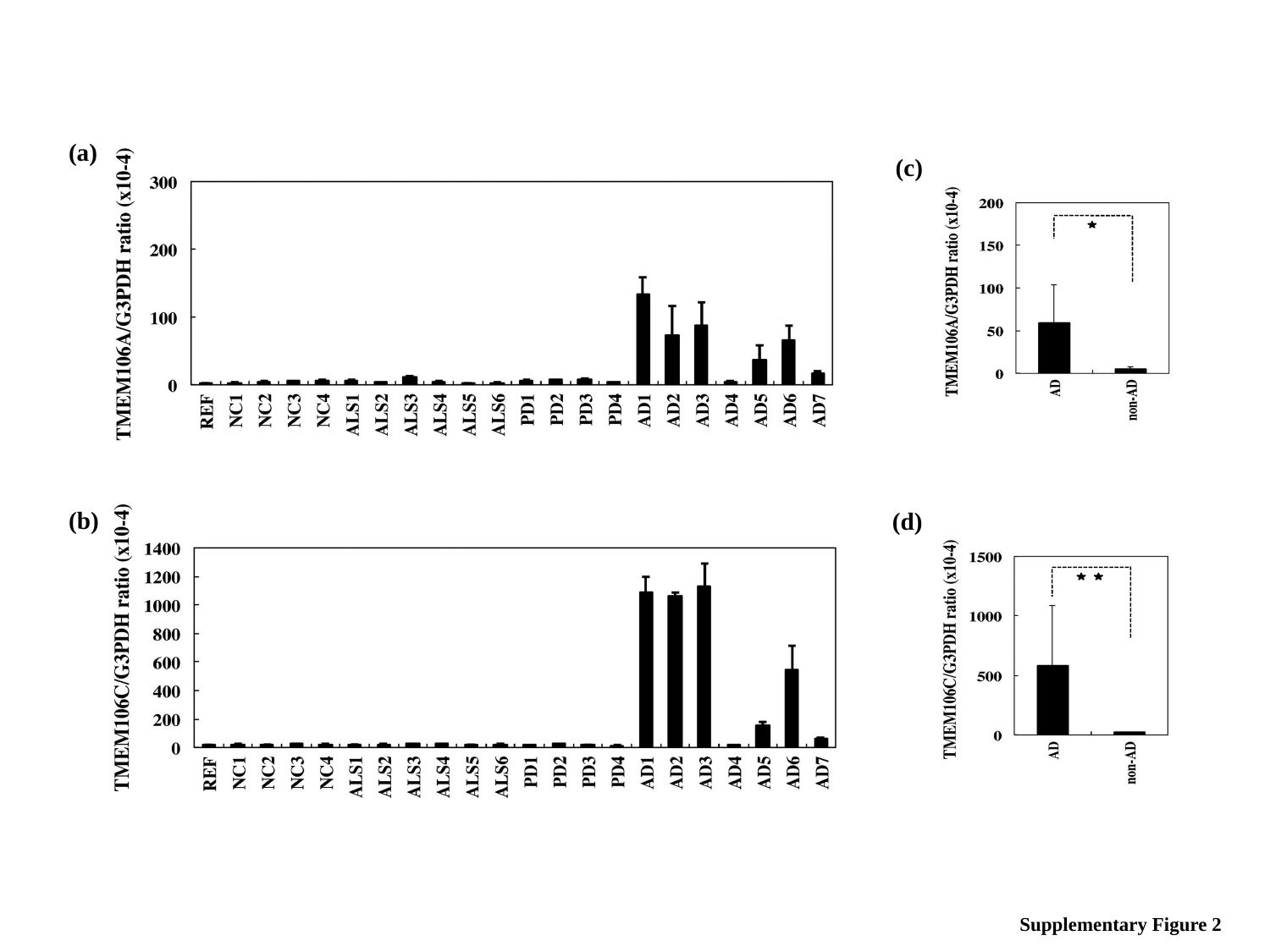

(a)
(c)
(b)
(d)
Supplementary Figure 2

Supplement: Additional file 2: Figure S2 — Showing elevated expression of TMEM106A and TMEM106C mRNA in AD brains. The TMEM106A and TMEM106C mRNA expression levels were studied by qPCR in human brain tissues derived from a REF, four NC cases, six ALS cases, four PD cases, and seven AD cases. The expression levels were standardized against those of G3PDH. (a) TMEM106A. (b) TMEM106C. (c) Difference in TMEM106A levels between AD and non-AD cases. *P = 0.0002 by Student’s t test. (d) Difference in TMEM106C levels between AD and non-AD cases. **P = 0.0005 by Student’s t test. [file alzrt247-S2.ppt]

## Slide 1
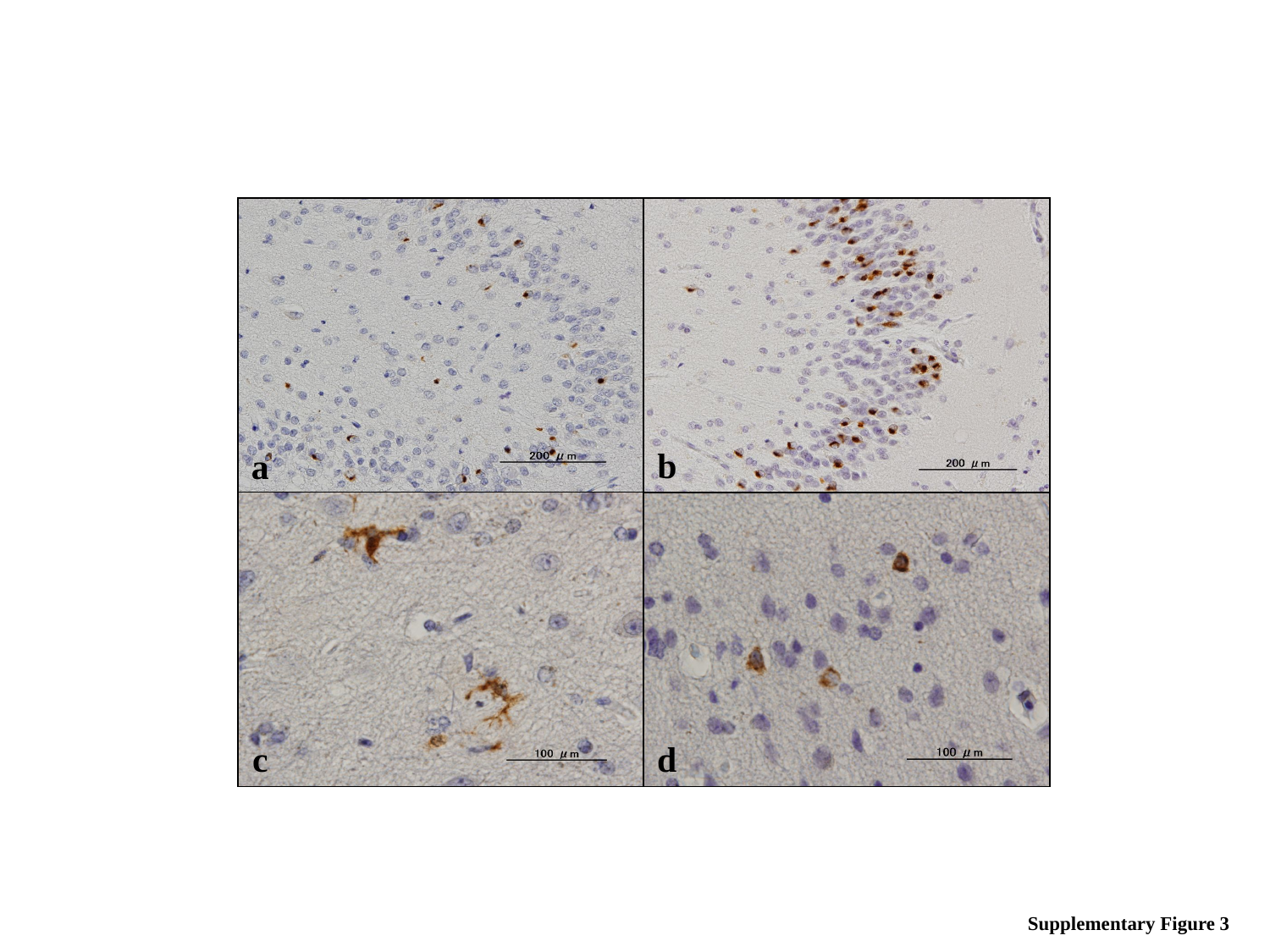

b
a
d
c
Supplementary Figure 3

Supplement: Additional file 3: Figure S3 — Showing pTDP-43 immunoreactivity in AD and non-AD brains. The expression of phosphorylated TDP-43 (pTDP-43) immunoreactivity was studied in six AD brains and 13 non-AD brains presented in Table 1 by immunohistochemistry using anti-pS409/410 TDP-43 antibody. (a) AD, the hippocampal granule cell layer, neuronal cytoplasmic staining; (b) ALS, the hippocampal granule cell layer, neuronal cytoplasmic staining; (c) AD, the frontal cortex, microglial cytoplasmic staining; (d) ALS, the frontal cortex, neuronal cytoplasmic staining. [file alzrt247-S3.ppt]

## Slide 1
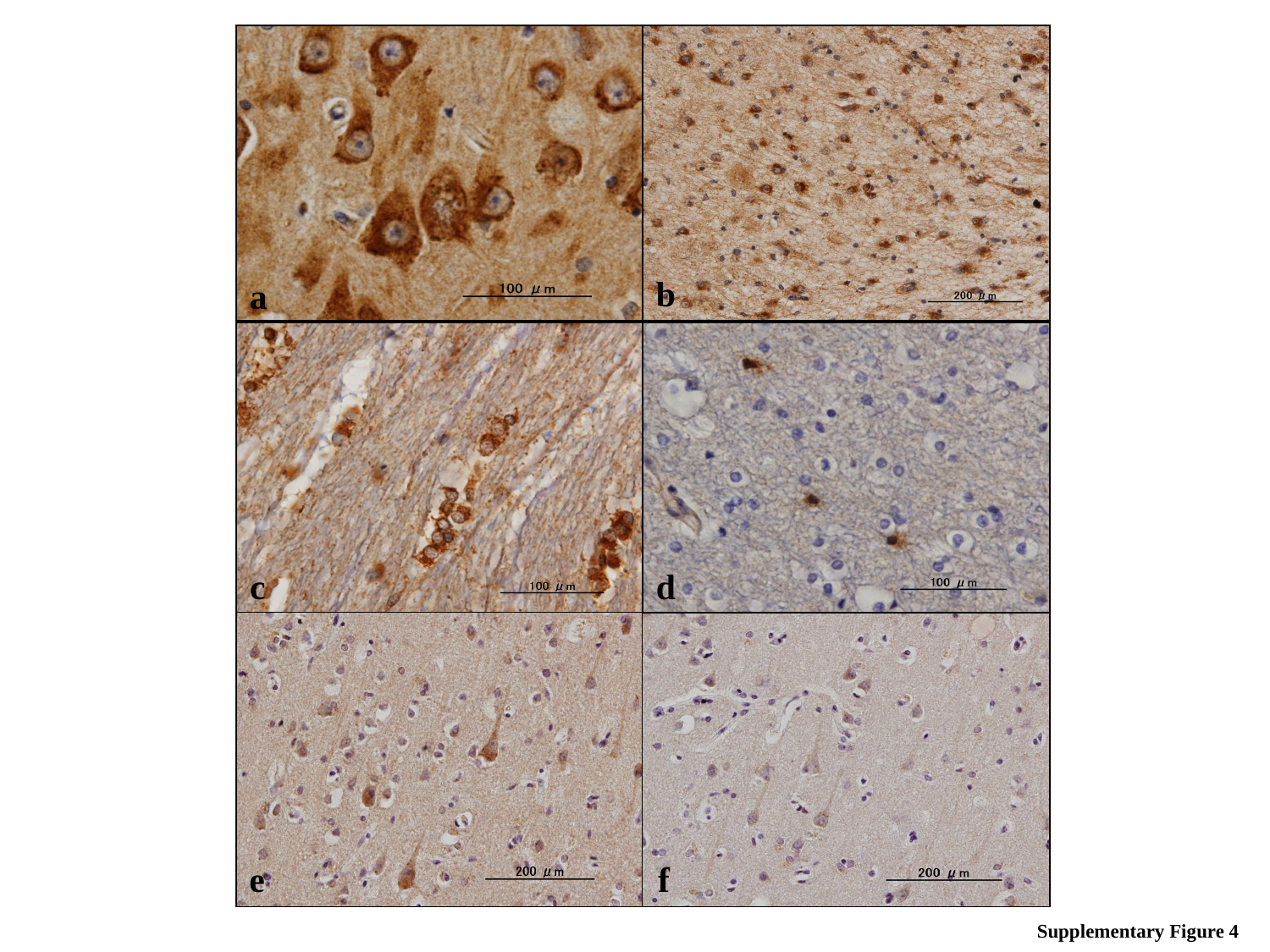

b
a
d
c
f
e
Supplementary Figure 4

Supplement: Additional file 4: Figure S4 — Showing TMEM106B and PGRN immunoreactivities in AD and non-AD brains. The expression of TMEM106 and PGRN immunoreactivities was studied in six AD brains and 13 non-AD brains presented in Table 1 by immunohistochemistry using the A303-439A antibody. (a) TMEM106B, AD, the hippocampal CA1 region, vacuoles of granulovacuolar degeneration (GVD) devoid of staining; (b) TMEM106B, AD, the hippocampal molecular layer, intense astroglial cytoplasmic staining; (c) TMEM106B, AD, the periventricular white matter, intense oligodendroglial cytoplasmic staining; (d) PGRN, AD, the frontal white matter, intense microglial cytoplasmic staining; (e) TMEM106B, PD, the frontal cortex, moderate/intense neuronal cytoplasmic staining; (f) TMEM106B after absorption of the antibody, same region as (e), diminished neuronal cytoplasmic staining. [file alzrt247-S4.ppt]

## Slide 1
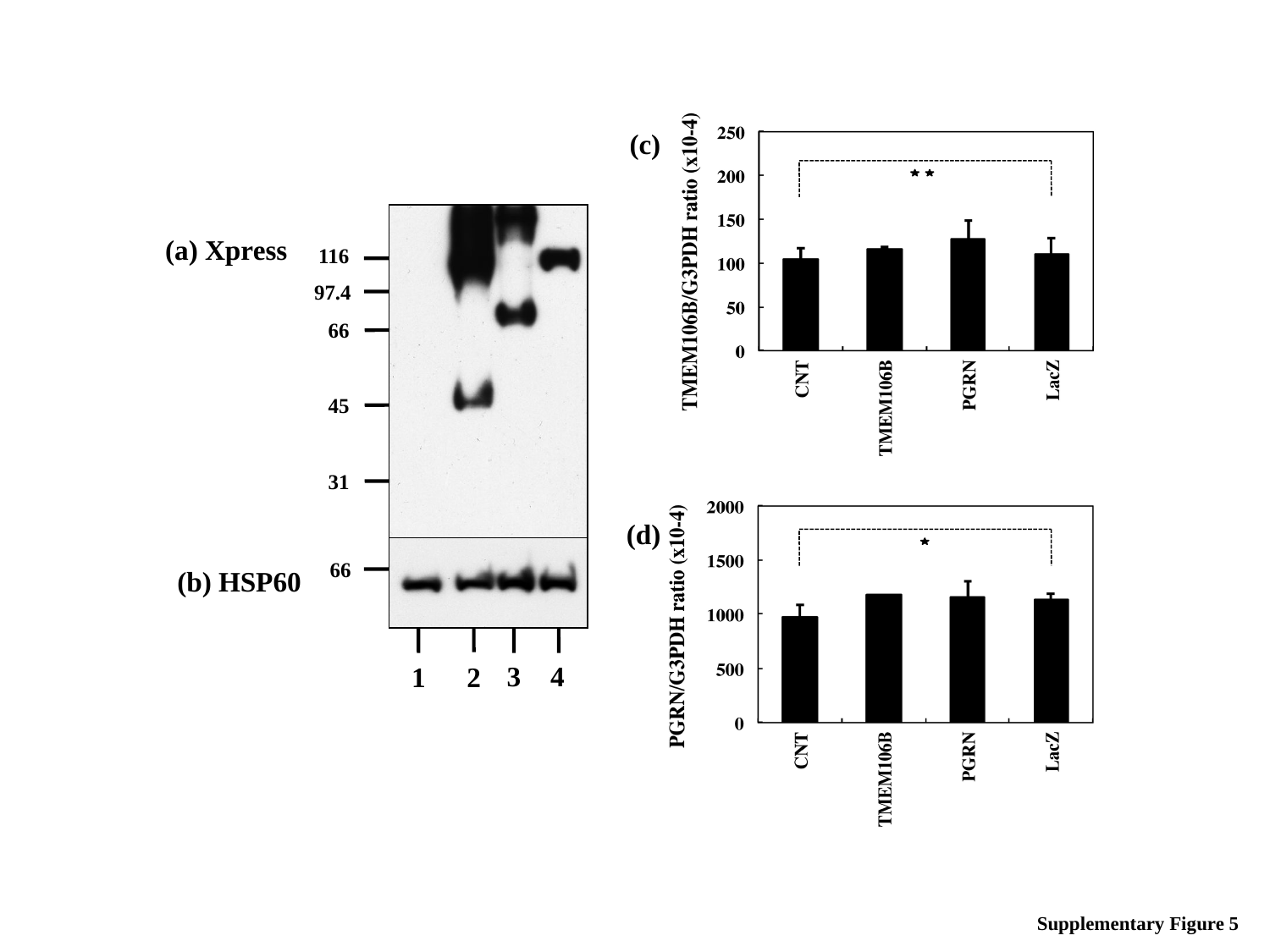

(c)
(a) Xpress
116
97.4
66
45
31
(d)
66
(b) HSP60
3
4
2
1
Supplementary Figure 5

Supplement: Additional file 5: Figure S5 — Showing overexpression of TMEM106B or PGRN did not alter PGRN or TMEM106B mRNA expression levels in SK-N-SH neuroblastoma cells. SK-N-SH neuroblastoma cells expressing Xpress-tagged recombinant proteins were processed for western blot and qPCR. Immunoblot of (a) Xpress and (b) HSP60, an internal control for protein loading. Lanes represent the protein of (1) untransfected cells and the cells expressing (2) TMEM106B, (3) PGRN, and (4) LacZ tagged with Xpress. mRNA expression levels of (c) TMEM106B and (d) PGRN in SK-N-SH cells exposed to Lipofectamine 2000 alone (CNT) and following expression of TMEM106B, PGRN, and LacZ proteins tagged with Xpress. (Single star indicates P = 0.1204 by one-way ANOVA, while double star indicates P = 0.4726 by one-way ANOVA). [file alzrt247-S5.ppt]
